# Optical Coherence Tomography Angiography as a Noninvasive Assessment of Cerebral Microcirculatory Disorders Caused by Carotid Artery Stenosis

**DOI:** 10.1155/2021/2662031

**Published:** 2021-07-05

**Authors:** Xinyue Li, Shiyi Zhu, Sujun Zhou, Yanwei Zhang, Yiheng Ding, Bingjie Zheng, Pei Wu, Yan Shi, Hong Zhang, Huaizhang Shi

**Affiliations:** ^1^Eye Hospital, First Affiliated Hospital of Harbin Medical University, China; ^2^Key Laboratory of Basic and Clinical Research of Heilongjiang Province, China; ^3^Department of Neurosurgery, First Affiliated Hospital of Harbin Medical University, China

## Abstract

**Purpose:**

Using retinal optical coherence tomography angiography (OCTA), we aimed to investigate the changes in important indicators of cerebral microcirculatory disorders, such as the properties of the radial peripapillary capillaries, vascular complexes, and the retinal nerve fiber layer, caused by carotid stenosis and postoperative reperfusion.

**Methods:**

In this prospective longitudinal cohort study, we recruited 40 carotid stenosis patients and 89 healthy volunteers in the First Affiliated Hospital of Harbin Medical University (Harbin, China). Eyes with ipsilateral carotid stenosis constituted the experimental group, while the fellow eyes constituted the contralateral eye group. Digital subtraction angiography, CT perfusion imaging (CTP), and OCTA examinations were performed in all subjects. The vessel density of the radial peripapillary capillaries (RPC), superficial retinal vascular complexes (SVC), deep vascular complexes (DVC), choriocapillaris (CC), and the thickness of the retinal nerve fiber layer (RNFL) were assessed. Propensity-matched analysis was undertaken to adjust for covariate imbalances. Intergroup comparative analysis was conducted, and the paired sample *t*-test was used to evaluate the preoperative and postoperative changes in OCTA variables.

**Results:**

The ocular vessel density in the experimental group was significantly lower than that in the control group (RPC: 55.95 vs. 57.24, *P* = 0.0161; SVC: 48.65 vs. 52.22, *P* = 0.0006; DVC: 49.65 vs. 57.50, *P* < 0.0001). Participants with severe carotid stenosis have reduced contralateral ocular vessel density (RPC 54.30; SVC 48.50; DVC 50.80). Unilateral stenosis removal resulted in an increase in vessel density on both sides, which was detected by OCTA on the 4^th^ day (RPC, *P* < 0.0001; SVC, *P* = 0.0104; DVC, *P* = 0.0104). Moreover, the ocular perfusion was consistent with that established by CTP.

**Conclusion:**

OCTA can be used for sensitive detection and accurate evaluation of decreased ocular perfusion caused by carotid stenosis and may thus have the potential for application in noninvasive detection of cerebral microcirculation disorders. This trial is registered with NCT04326842.

## 1. Introduction

Carotid artery stenosis (CAS) is one of the major causes of cerebral ischemic stroke, and its early detection is of considerable significance for stroke prevention [1, 2]. Overall, the prevalence of asymptomatic carotid stenosis in the general population ranges from 1.6% to 5.7% [3]. Delays in timely diagnosis and treatment aggravate patients' cognitive decline and substantially increase the risk of falling [4]. Due to the high CAS-inflicted disability rates and the great economic burden imposed on society, screening for abnormalities preceding the occurrence of such severe complications is of critical global importance.

Despite the rapid evolution of magnetic resonance imaging (MRI) perfusion techniques and the wide availability of perfusion single-photon emission computed tomography (SPECT), PET perfusion imaging with radiolabeled water is still considered the gold standard in cerebral perfusion evaluation with imaging [5]. Due to its high price and radiation risk, this approach cannot be used for large-scale early screening of cerebral ischemia. Thus, CT perfusion imaging (CTP) is commonly used in the clinic. However, most carotid artery stenosis patients are unable to tolerate CTP because of their poor systemic vascular condition. Therefore, a simple, noninvasive, and inexpensive screening tool for carotid artery stenosis is crucial.

The ophthalmic artery is an important branch of the internal carotid artery. The retinal and cerebral arterioles originate from the same germ layer and have common anatomical and physiological characteristics [6]. Therefore, retinal microvascular abnormalities are considered to reflect the degree of cerebral microvascular disease [7]. Several studies [8–11] have confirmed that ocular manifestations can reflect the changes of carotid vessels; however, the potential of previously employed examination methods is limited. For example, a risk of allergy to contrast media exists in retinal angiography [12]. Moreover, other methods, such as retinal thickness [11] and fundus photography [10], do not possess adequate accuracy for the observation of tiny blood vessels. Optical coherence tomography angiography (OCTA) is a new technology that provides high-resolution retinal vascular images suitable for direct observation of 5 *μ*m capillaries. Although this noninvasive, rapid, and accurate technique can automatically and quantitatively measure the ocular vessel density, no published reports exist on the use of OCTA for cerebral perfusion prediction. The purpose of this study was to establish the relationship between retinal blood perfusion detected by OCTA and cerebral perfusion.

## 2. Methods

### 2.1. Study Design and Participants

This single-institutional, prospective, cohort, exploratory, observational clinical trial was designed in compliance with the Declaration of Helsinki. The study protocol was approved by the Ethics Committee of the First Affiliated Hospital of Harbin Medical University (Harbin, China) and was registered at the International Clinical Trials Register (http://www.clinicaltrials.gov. NCT04326842). All subjects signed informed consent forms prior to their inclusion in the study.

We recruited patients with carotid artery stenosis from the neurosurgical ward and healthy controls from physical examination centers between April 2020 and July 2020.

The following eye examinations had to be completed in all participants: visual acuity (VA), diopter measurement using automated optometry, intraocular pressure (IOP) measurement by a noncontact tonometer, slit-lamp biomicroscope, fundus examination, and OCTA. All subjects were examined by ultrasound to determine whether the bilateral carotid arteries were stenosed. Controls were confirmed without carotid artery stenosis. Patients with stenosis were further preoperatively examined by digital subtraction angiography (DSA) to determine the degree of carotid artery stenosis. The grade of carotid stenosis was evaluated using DSA in accordance with the North America Symptomatic Carotid Endarterectomy Trial (NASCET) criteria [13]. The degree of carotid artery stenosis was graded as moderate (50%–70%) and severe (≥70%). The system medical history and family history of each subject were also recorded.

### 2.2. Exclusion Criteria

Subjects were excluded if the following criteria were met: (1) age < 18 years; (2) IOP > 21 mmHg; (3) VA > logMAR 1.0; (4) spherical equivalent (SE) > −6.00 D; (5) other serious eye diseases which may affect OCTA results, including diabetic retinopathy, hypertensive retinopathy, retinal vascular occlusion, age-related macular degeneration, and uveitis; (6) major intraocular surgery performed in the past six months or a history of laser photocoagulation or intravitreal injection; (7) glaucoma or first-degree relatives with a history of glaucoma; and (8) any disease that might cause poor scan quality (image quality < 6).

### 2.3. OCTA: Technique and Clinical Applications

**Figure 1:**
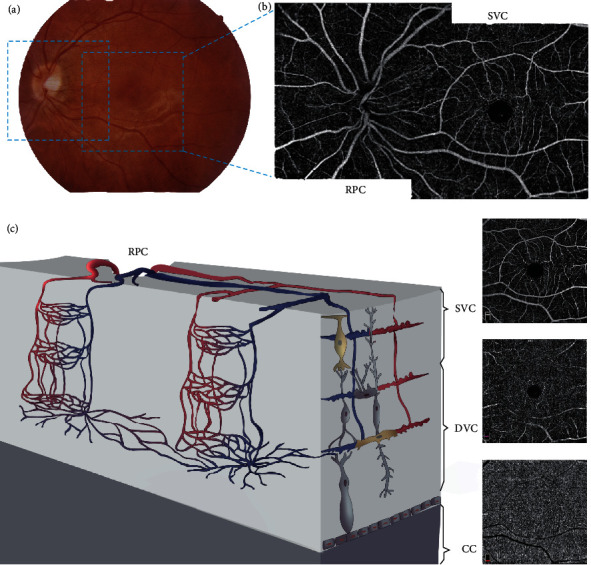
Retinal vascular plexuses and interconnecting layers. (a) Color fundus photograph demonstrates two 6 × 6 mm regions of scan (blue square); (b) the OCTA en face images are arrayed from the most superficial on top to the deepest at the bottom; (c) the cartoon depicts the anatomical relationships between arterial and venous systems in the two vascular plexuses and the interconnecting layers. RPC: radical peripapillary capillaries; SVC: superficial vascular complexes; DVC: deep vascular complexes; CC: choriocapillaris.

The same experienced examiner used AngioVue® OCTA (Optovue, Inc., Fremont, CA, USA) to scan all eyes with 6 × 6 mm of macular area and 4.5 × 4.5 mm of optic nerve area without mydriasis to obtain microvascular images of the eyes. Each scan was repeated three times to obtain an optimal image quality. The instrument was operated at a central wavelength of 840 nm and a speed of 68,000 A-scans per second, with each B-scan having 245 A-scans in both the horizontal and vertical directions. The microangiography composite algorithm analyzes the changes of the complex signal (the intensity and phase changes are included in the continuous B-scan at the same position) and then generates the microvascular image. As can be seen in Figure 1, based on the distribution and shape of the retinal blood vessels, the retinal microvascular system was divided into radial peripapillary capillaries (RPC), superficial vascular complexes (SVC), deep vascular complexes (DVC), and choriocapillaris (CC). OCTA can also measure the thickness of the retinal nerve fiber layer (RNFL). The image analysis software of OCTA equipment was used for automatic analysis of the image, and a series of parameters such as vessel density (VD) and retinal thickness were calculated (Supplementary Figure 1). This version of AngioVue® software (RTVue XR version 2018.1.0.43, Optovue, Inc., Fremont, CA, USA) can remove the projection artifacts on the deep retina through 3D projection artifact removal (3D PAR) technology, facilitating clearer visualization at all depths of the vascular structures in the enface images and B-scans. VD was defined as the proportion of the blood flow signal detected by OCTA to the corresponding area. The numerical value was calculated automatically by the OCTA software.

### 2.4. CTP: Technique and Clinical Applications

All patients were examined pre- and postoperatively by CTP to determine the cerebral blood flow perfusion of patients with carotid artery stenosis. CTP imaging was performed on a Philips 256-slice CT scanner (Philips Brilliance iCT). Cerebral perfusion scan mode was employed with a tube voltage of 120 kV and a tube current of 150 mA. The scanning time was 50 s, the layer thickness was 5 mm, and the coverage was 12 cm. Lobitridol injection (350 mg/mL) was used as the contrast medium. The total amount was 100 mL, and the applied injection flow rate was 5 mL/min. We obtained 400 CTP images by scanning, which were then transferred into a special postprocessing workstation. Postprocessing of the CTP scans was performed using commercially available software with an Extended Brilliance Workspace (version 4.5.4, Philips Healthcare). The images were analyzed by two experienced neuroimaging diagnostics. In the perfusion image, the central layer of the semioval center layer, the body of the lateral ventricle layer, and the basal ganglia layer were considered as the region of interest (ROI) to avoid the inclusion of calcifications and old infarcted tissues as far as possible. The frontal, temporal, and parietal lobes of the middle cerebral artery were drawn by hand in the same area as the ROI. The cerebral blood flow (CBF), cerebral blood volume (CBV), mean transit time (MTT), and peak time (TTP) of the lesion side and the contralateral corresponding area were measured by mirror symmetry with the midline. The pre- and postoperative data obtained were quantitatively and qualitatively analyzed and compared. Each level was measured three times; finally, the average value was taken to ensure that the final selected ROI data were reliable and consistent.

### 2.5. Interventional Procedure

The patients in the experimental group were treated with carotid endarterectomy (CEA) or carotid artery stenting (CAS). Their surgical indications, surgical procedures, and surgical norms were performed in compliance with the AHA guidelines [14] and “Rutherford's Vascular Surgery.” [15].

### 2.6. Follow-Up Assessments

Before discharge, OCTA and CTP examinations were repeated in all patients on the 4^th^ day after the operation. This information was also collected and recorded. The hypothesis of blinding was considered during the study design: technicians were blinded to the patient's condition before and after surgery.

### 2.7. Statistical Analysis

This study was purely exploratory; thus, no prospective sample size calculation was conducted.

Categorical variables are presented as percentages, continuous variables as mean ± SD, and skewed variables as median (boundaries of interquartile range, IQR). Propensity score-matched (PSM) analysis was performed using a multivariable logistic regression model based on age, gender, tobacco use, alcohol intake, and diabetes mellitus status. Participants were matched in pairs using 1 : 1 greedy nearest neighbor matching, resulting in the formation of 31 matched pairs in each group.

The clinical characteristics of the groups were compared using the *χ*^2^ test for categorical variables, paired *t*-test for normally distributed continuous variables, and Wilcoxon rank-sum tests for nonnormally distributed continuous variables. Paired sample *t*-tests were employed to assess and compare the preoperative and postoperative changes in OCTA variables.

Fisher's exact probability test was applied to verify the *P* value when 25% of the theoretical frequency was lower than 5. All tests were bilateral, with statistical significance at *P* < 0.05. All statistical analyses were performed using SPSS software (v25, IBM, Armonk, NY, USA), and Prism 7 (v7.02, GraphPad, La Jolla, CA, USA) was employed for graph generation.

## 3. Results

### 3.1. Characteristics of the Participants

**Figure 2:**
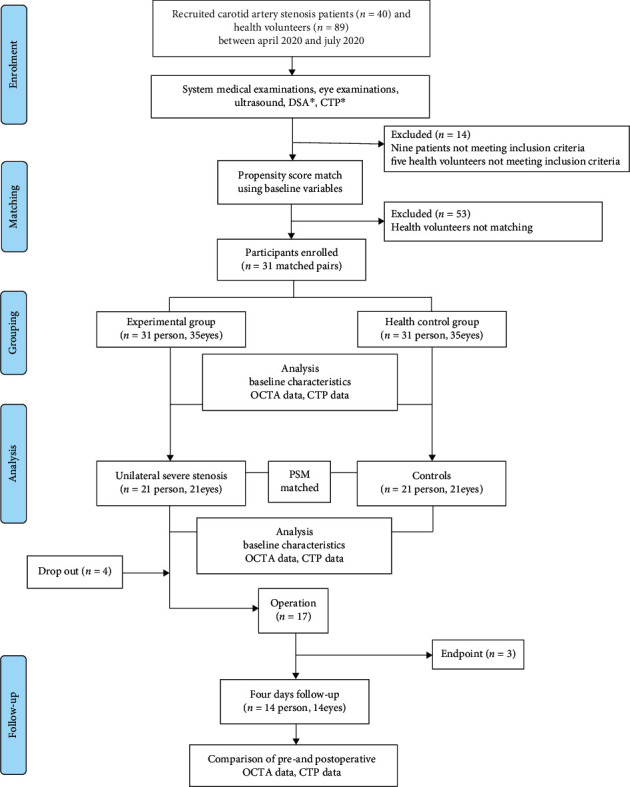
Flow chart of patient recruitment into the study. ^∗^DSA CTP only for patients.

A total number of 129 participants (40 patients and 89 healthy volunteers) were recruited between April 2020 and July 2020 (Figure 2). Fourteen participants were excluded for not meeting the inclusion criteria. After PSM analysis, 31 matched pairs were obtained (31 in the experimental group and 31 in the healthy control group). Of the 31 patients in the experimental group, 14 cases of unilateral severe stenosis or occlusion were followed up on the 4^th^ day postoperatively.

After 1 : 1 PSM adjustment, baseline demographic and clinical variables were well balanced between the two groups (Table 1).

### 3.2. RPC, SVC, DVC, and CC Vessel Densities

**Figure 3:**
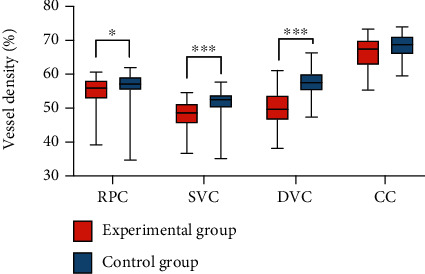
Vessel density of the stenotic side of the eye. The vessel density of the stenotic side of the eye in the experimental group was lower than that in the control group. RPC: radical peripapillary capillaries; SVC: superficial vascular complexes; DVC: deep vascular complexes; CC: choriocapillaris.

The RPC, SVC, and DVC vessel densities in the patients were significantly lower than those in the healthy controls, with statistically significant differences (*P* = 0.0161; 0.0006; <0.0001, Table 1). Although no statistical difference was available in CC between the two groups, the choriocapillaris VD of the diseased side was generally lower than that of the control group (Figure 3).

### 3.3. Bilateral Changes Caused by Unilateral Carotid Stenosis

**Figure 4:**
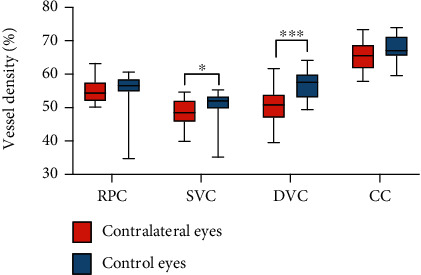
Vessel density of the contralateral eyes with severe unilateral carotid artery stenosis. The vessel density of the contralateral eyes with severe unilateral carotid artery stenosis in the experimental group was lower than that of the control eyes. RPC: radical peripapillary capillaries; SVC: superficial vascular complexes; DVC: deep vascular complexes; CC: choriocapillaris.

Of the 31 patients in the experimental group, 21 had unilateral severe stenosis or occlusion. Twenty-one controls that matched the general condition of the control group were selected by the PSM tendency score. The retina vessel densities of the two groups were then compared. The results showed that although the contralateral carotid artery had no stenosis in patients with severe unilateral carotid artery stenosis and occlusion, the blood supply of the contralateral eyes was reduced, and the SVC and DVC vessel densities were significantly lower than those of the control eyes, with statistically significant differences (*P* = 0.0123; 0.0007, Table 2). As can be seen in Figure 4, the RPC and CC vessel densities of the contralateral eyes were generally slightly lower than those of the control eyes.

### 3.4. OCTA and Relationship with CTP

**Figure 5:**
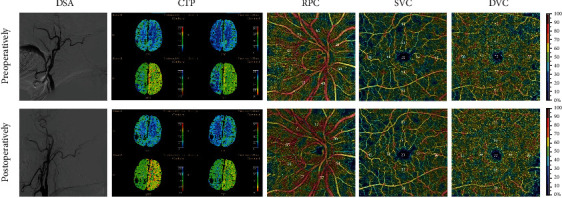
Brain and eye perfusion in the patients with moderate and severe carotid stenosis. Color bars represent vessel density; brighter colors indicate higher values. RPC: radical peripapillary capillaries; SVC: superficial vascular complexes; DVC: deep vascular complexes; CC: choriocapillaris; RPC: radical peripapillary capillaries; SVC: superficial vascular complexes; DVC: deep vascular complexes; CC: choriocapillaris.

In this study, we found that patients with poor cerebral perfusion caused by severe carotid artery stenosis or occlusion also had poorer retinal vessel density than patients with moderate carotid artery stenosis (Figure 5).

### 3.5. Comparison of Pre- and Postoperative OCTA and CTP

**Figure 6:**
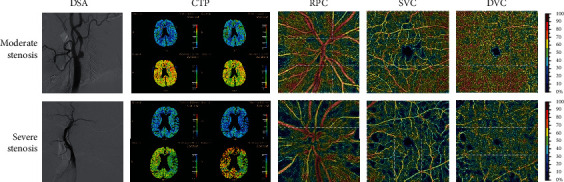
Preoperative (top row) and postoperative (bottom row) images of a patient with carotid artery occlusion. Compared with the preoperative OCTA image, there were more brightly colored areas (suggesting an increase in the blood flow density in this area), and the value of VD on the image was also higher. These changes were not observed in the CTP's image. RPC: radical peripapillary capillaries; SVC: superficial vascular complexes; DVC: deep vascular complexes; CC: choriocapillaris.

Of the 21 patients with unilateral severe stenosis, 14 were followed up on the 4^th^ day after the operation. The characteristics of their condition are presented in Table 3. After the operation, with the improvement of cerebral perfusion, the ocular blood supply of the operated side was significantly improved (PRC, *P* < 0.0001; SVC, *P* = 0.0104; DVC, *P* = 0.0104; RNFL, *P* < 0.0001). Meanwhile, the condition of the contralateral eye had also improved. This significant change is visible in Figure 6.

## 4. Discussion

Our study confirmed and explained the mechanism by which carotid artery stenosis or occlusion affects the ocular blood supply. OCTA can sensitively detect subtle changes in the ocular blood supply in the early stage of carotid artery stenosis and after CEA or CAS surgery. We confirmed the relationship between ocular microcirculation and cerebral perfusion. Therefore, OCTA may be used as a noninvasive quantitative screening method for carotid artery stenosis or occlusion.

Atherosclerosis is a systemic vascular disorder involving multiple arterial beds, including carotid and coronary arteries [16]. Detection of atheroma plaque by noninvasive techniques in the easily accessible carotid artery emerged as a surrogate marker for advanced atherosclerosis in other vascular beds, including coronary arteries [17]. The carotid artery is the earliest involved blood vessel in the development of atherosclerosis [18]. Carotid artery stenosis is defined by the narrowing of the carotid arteries and commonly caused by atherosclerosis, potentially leading to ischemic disease. All ocular vessels receive their blood supply from the ophthalmic artery, a branch of the internal carotid artery. On the other hand, systemic diseases such as diabetes and hypertension can cause chronic damage to vessels, including carotid arteries and ocular vessels. Therefore, from the anatomical point, stenosis or occlusion of the proximal vessel can naturally affect the blood supply of the distal branch. From the pathological point, carotid artery and ocular vascular density damage also have many similarities.

Increasingly more scientific attention has been paid to microvessel damage in cerebrovascular disease research. However, microvascular lesions cannot be directly observed using traditional examination methods. Moreover, due to the radiation trauma caused by the examination, it is not feasible to check repeatedly and monitor the disease. Previous studies have shown that carotid artery stenosis causes morphological changes in certain eye characteristics, including an abnormal diameter of the retinal vessels [10], thinning of RNFL [19], and thinning of the choroid [20]. Several investigations have revealed that retinal vessels can be used as a noninvasive observation object for reliable evaluation of the cerebral microvascular system [12, 21, 22]. The birth of the OCTA technology has enabled the observations not only of the small blood vessels with an approximate size of 250 *μ*m but also of capillaries with a size of 5 *μ*m. This increased visibility facilitates the implementation of accurate observations of the microvascular system of the brain. OCTA is not only simple and fast to operate but also radiation-free. Furthermore, this approach does not require the administration of intravenous dye injection, and thus, it is suitable for early detection of ischemia and follow-up detection. The automatic blood flow measurement function of Optovue has been certified by FDA, and its objectivity has been widely recognized. In normal people, macular vessel density is extremely stable and can reflect the amount of circulatory perfusion. Hence, macular vessel density has become an excellent tool to measure the impact of various retinopathies and even systemic microcirculation diseases, such as diabetes [23], hypertension [24], cerebral small vessel disease [25], Alzheimer's disease [26], and migraine [27].

Our first major finding was that the ocular vessel density on the diseased side in unilateral moderate carotid artery stenosis was lower than that in normal controls (Table 1, Figure 3). This result is in line with the findings of previous studies showing worse retinal vascular parameters in subjects with carotid artery stenosis. For example, thinner retinal vessel caliber, lower venous responses to flicker stimulation, and decreased central retinal arteriolar equivalent [10] were reported to be associated with carotid artery stenosis. However, manually measured retinal vasculature measurements were employed in these previous studies, which are subjective. Using OCTA, we easily examined the microvasculature down to the capillary level. In a previous study, the OCTA results showed that the flow density in the superficial retinal OCT angiogram of the macula in patients with CAS was significantly lower than that in healthy controls (study group: 48.52 ± 4.46; control group: 51.88 ± 2.70; *P* = 0.003) [28]. This finding is consistent with our research results.

Carotid artery stenosis exceeding 70% not only affected the ocular blood supply of the ipsilateral side but also reduced the ocular vessel density of the contralateral side (Table 2, Figure 4). After CEA or CAS operation, the ocular vessel densities on both operated and nonoperated sides increased. Previous studies obtained similar results, in which unilateral carotid revascularization improved the cerebral perfusion and metabolism not only of the ipsilateral cerebral hemisphere but also of the contralateral cerebral hemisphere [4, 29, 30]. In an earlier examination, Lee et al. recruited 20 patients with severe carotid stenosis, and OCTA was performed before CAS and one month after that. The vessel density on the operated side of the DVC increased significantly after stent implantation (*P* = 0.010). Interestingly, the vessel densities in the SVC (*P* = 0.028) and DVC (*P* = 0.034) were significantly higher after stent implantation on the nonoperated side as well. Evidence shows that the bilateral ocular blood flow [31] and the retinal electrophysiological response [32] were improved postoperatively. Unfortunately, this phenomenon has not been well explained to date. Here, we present two hypotheses concerning its possible mechanism. First, from a physiological point of view, when the blood supply to one side of the cerebral hemisphere is decreased, the body establishes a contralateral collateral circulation to balance the global cerebral perfusion. Liu et al. measured the direction of the ophthalmic artery flow and bilaterally in the internal carotid arteries in a group of 116 patients with unilateral carotid artery stenosis and found that the direction was reversed in 36% of the patients [33]. In another study, Pienimaki et al. observed that patients with severe carotid stenosis were four times more likely to develop collateral circulation than patients with nonstenosis and moderate stenosis [34]. In our study, we established the presence of collateral circulation in 44% of patients with unilateral severe carotid artery stenosis or occlusion by DSA (Supplementary Figure 2). Second, patients with carotid artery stenosis are at a high risk of arteriosclerosis development. Although the nonoperative side vessels were not stenosed, they might have been affected by arteriosclerosis, which could also have influenced the blood flow density. When we selected patients in the experimental and control groups, we matched their baseline data, which might have affected the blood flow density, whereas the conditions in terms of hypertension, diabetes, smoking, and drinking in the two groups were similar. By this matching, we minimized the impact of noncarotid stenosis factors on the results. Our findings provide evidence that unilateral stenosis or occlusion results in a decrease in the blood supply to the contralateral eye, while unilateral surgery improves the contralateral eye condition. Such changes cannot be observed by traditional examination approaches, whereas OCTA can be used repeatedly for monitoring the perfusion of different regions of the left and right retinal circulation longitudinally.

Besides vessel density, we were also interested to elucidate the impact of carotid blood flow on RNFL. The RNFL is composed of axons of ganglion cells. Earlier studies have established that the thickness of RNFL decreased associate with the development of cognitive impairment, stroke, and other brain diseases [22, 35]. We found that the thickness of RNFL increased (Table 3) on both sides after CEA or CAS operation. This outcome indicates that relieving carotid artery stenosis or occlusion may also influence RNFL. Previous investigations did not detect such a significant difference in RNFL [19, 36] as the resolution of EDI-OCT was not as high as that of OCTA.

Choroidal blood flow accounts for 90% of the ocular blood flow [37]. However, whether carotid artery stenosis has an impact on the choroidal blood flow remains unknown. Using the automatic stratification and quantification features of OCTA, we objectively measured the value of the choroidal vessel density (Tables 1 and 2) and found that its values in the experimental group were slightly lower than those in normal subjects. Because of the large choroidal blood low, slight changes may not have had a statistically significant difference in the limited number of observed cases in our study. The significance of the overall difference may be increased by the inclusion of more patients.

Our research has some limitations. First, it was conducted over a relatively short period of three months. Further investigations are required to establish the potential long-term existence of these early beneficial effects. Second, although we prospectively recruited well-characterized subjects, this study was exploratory, with relatively a small sample size. Further longitudinal studies with a larger sample size are needed to confirm our results. Third, despite the relatively weak correlation between OCTA and CTP results (Figures 5 and 6), we can still see the trend of improvement in the images of cerebral perfusion and eye perfusion after CEA or CAS operation. CTP is the ratio of the two sides, but the ROI area measurement is subjective. Therefore, OCTA is an objective and accurate method for retinal vessel density measurement. Nevertheless, further studies with larger sample sizes are needed to obtain additional evidence and clarify the relationship between OCTA and CTP.

## 5. Conclusions

In conclusion, patients with carotid artery stenosis showed a reduced vessel density in the RPC, SVC, and DVC layer when compared with healthy controls. In patients with severe carotid stenosis, unilateral CAS effectively improved the retinal microcirculation in both eyes. OCTA can detect noninvasive and rapid changes in the retinal microcirculation and, along with CTP, may help establish a new biomarker for predicting and monitoring cerebral perfusion changes in patients with carotid artery stenosis.

## Figures and Tables

**Table 1:** Characteristics of the study participants.

Characteristic	Experimental group	Control group	*χ* ^2^/*W*/*Ζ*	*P*
Age, median (y)	63	63	249.5	0.1495
Male, *n* (%)	27 (87.1%)	23 (74.2%)	-2.1082	0.0719
Tobacco, *n* (%)	15 (48.4%)	14 (45.2%)	0.0648	0.7991
Alcohol, *n* (%)	11 (35.5%)	6 (19.4%)	2.0261	0.1546
Hypertension, *n* (%)	28 (90.3%)	21 (67.7%)	4.7692	0.0586^a^
Diabetes, *n* (%)	6 (19.4%)	9 (29.0%)	0.7915	0.3737
*OCTA characteristic*				
RPC VD (%) Median (IQR)	55.95 (53.30-58.10)	57.24 (56.00-59.20)	5.7924	0.0161
SVC VD (%) Median (IQR)	48.65 (45.50-51.10)	52.22 (50.14-53.53)	11.785	0.0006
DVC VD (%) Median (IQR)	49.65 (46.50-53.70)	57.50 (53.80-59.85)	20.152	<.0001
CC VD (%) Median (IQR)	67.41 (62.90-69.92)	68.74 (65.91-71.11)	1.560365	0.1320

VD: vessel density; RPC: radical peripapillary capillaries; SVC: superficial vascular complexes; DVC: deep vascular complexes; CC: choriocapillaris. ^a^The *P* value was verified by Fisher's exact probability test.

**Table 2:** Ocular conditions of contralateral eyes with severe unilateral carotid artery stenosis and occlusion and healthy control eyes.

OCTA characteristic	Contralateral eyes	Control eyes	*Z*	*P*
RPC VD (%) Median (IQR)	54.30 (51.90-57.60)	57.07 (55.13-59.16)	2.7747	0.0958
SVC VD (%) Median (IQR)	48.50 (45.80-52.00)	52.22 (50.14-53.21)	6.2650	0.0123
DVC VD (%) Median (IQR)	50.80 (47.10-53.80)	56.13 (53.07-58.63)	11.621	0.0007
CC VD (%) Median (IQR)	65.50 (61.90-68.76)	67.02 (65.88-71.11)	1.5597	0.1188

VD: vessel density; RPC: radical peripapillary capillaries; SVC: superficial vascular complexes; DVC: deep vascular complexes; CC: choriocapillaris.

**Table 3:** Changes of blood supply to the eyes and brain before and after the operation.

OCTA characteristic	Operated side median (IQR)	*P*	Nonoperated side median (IQR)	*P*
RPC VD (%)	1.55 (0.40-2.60)	<0.0001	0.65 (-1.65-2.20)	0.4332
Median (IQR)				
SVC VD (%)	1.70 (0.40-2.80)	0.0104	0.00 (-2.10-4.60)	1.0000
Median (IQR)				
DVC VD (%)	4.00 (0.70-6.50)	0.0104	-1.55 (-3.60-4.30)	0.1691
Median (IQR)				
RNFL (*μ*m)	2.50 (2.00-11.00)	<0.0001	4.00 (0.50-6.50)	0.005
Median (IQR)				
rCBF	0.19 (0.02-0.31)	0.0011	—	—
△MTT	-0.22 (-0.46--0.16)	<0.0001	—	—

VD: vessel density; RPC: radical peripapillary capillaries; SVC: superficial vascular complexes; DVC: deep vascular complexes; RNFL: retinal nerve fiber layer; rCBF: the ratio of the values measured in the symptomatic hemisphere to those in the asymptomatic hemisphere for cerebral blood flow; △MTT: the absolute difference in mean transit time values between the symptomatic and the asymptomatic hemispheres.

## Data Availability

Data is available from the corresponding author on reasonable request.

## Supplementary Materials

Supplementary Materials Supplementary Figure 1: OCTA AngioRetina 6.0 report. Supplementary Figure 2: preoperative DSA images of patients with severe carotid stenosis. (A) Severe stenosis at the beginning of the left internal carotid artery of the patient; (B) a significant compensation was observed in the intracranial blood supply from the right internal carotid artery to the left cerebral hemisphere through anterior communication.
