# Supplementary material for: Optical Coherence Tomography Angiography as a Noninvasive Assessment of Cerebral Microcirculatory Disorders Caused by Carotid Artery Stenosis
Source: Dis Markers. 2021 Jul 5;2021:2662031. doi: 10.1155/2021/2662031 (PMC8277520; doi:10.1155/2021/2662031)
Supplement: Supplementary Materials — Supplementary Figure 1: OCTA AngioRetina 6.0 report. Supplementary Figure 2: preoperative DSA images of patients with severe carotid stenosis. (A) Severe stenosis at the beginning of the left internal carotid artery of the patient; (B) a significant compensation was observed in the intracranial blood supply from the right internal carotid artery to the left cerebral hemisphere through anterior communication. [file 2662031.f1.pdf]

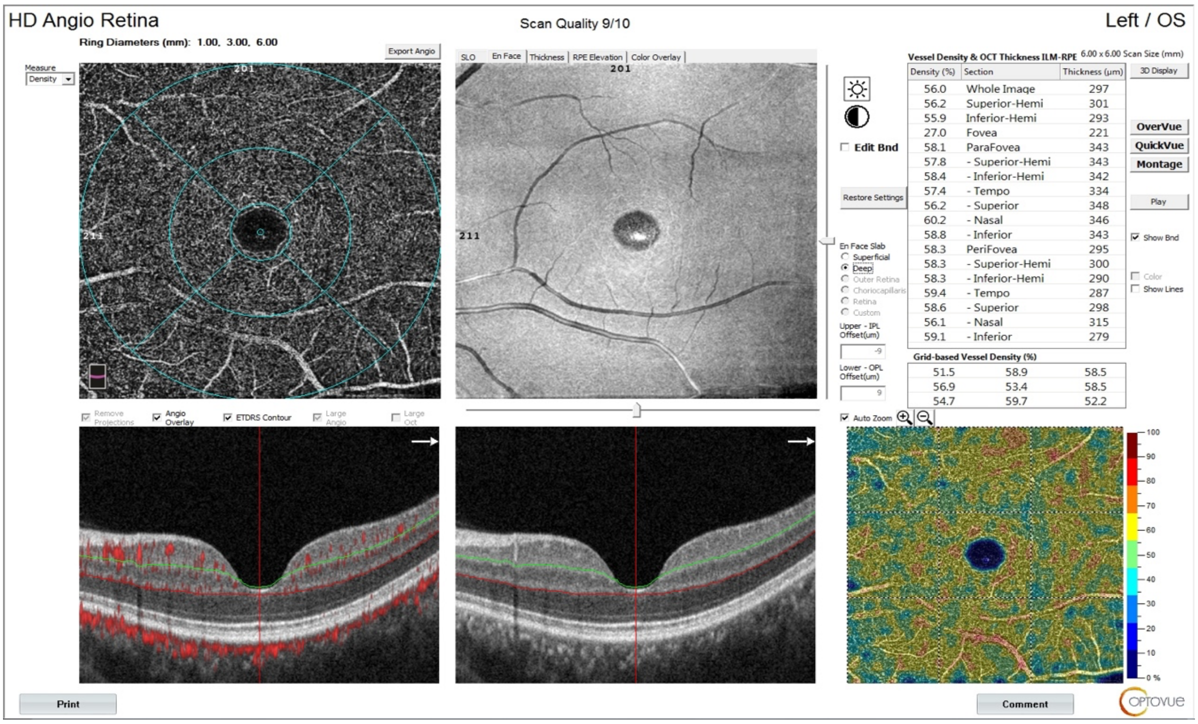

Supplementary Figure 1. OCTA AngioRetina 6.0 report

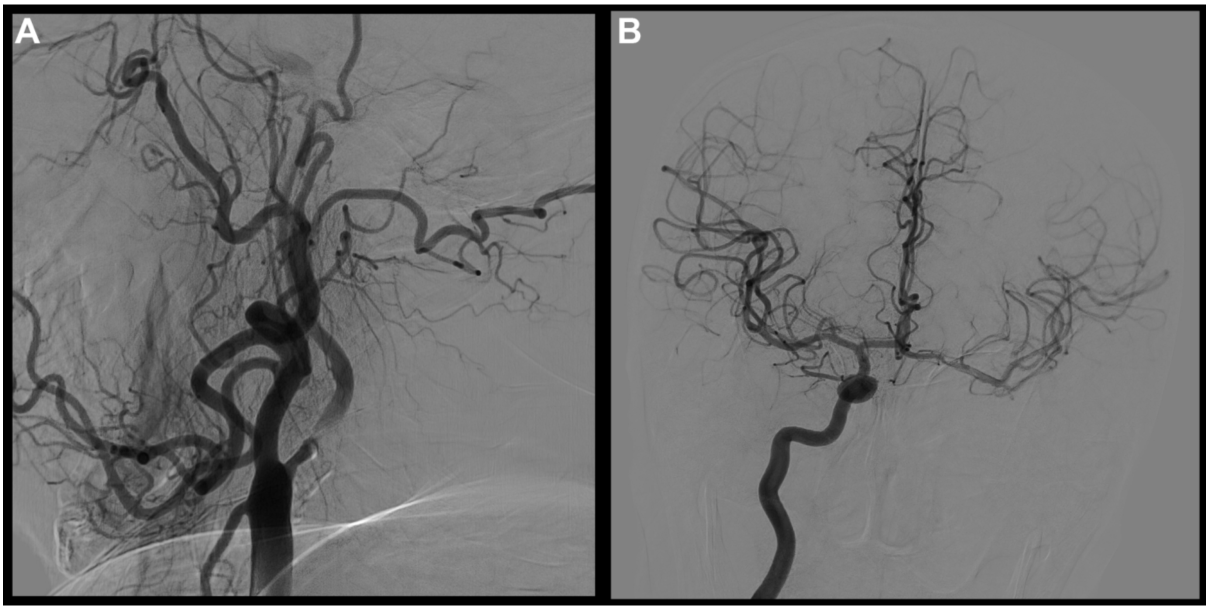

Supplementary Figure 2. Preoperative DSA images of patients with severe carotid stenosis. (A) Severe stenosis at the beginning of the left internal carotid artery of the patient; (B) A significant compensation was observed in the intracranial blood supply from the right internal carotid artery to the left cerebral hemisphere through anterior communication.
